# Childhood theory of mind does not predict psychotic experiences and social functioning in a general population sample of adolescents

**DOI:** 10.1371/journal.pone.0213165

**Published:** 2019-02-28

**Authors:** Laura A. Steenhuis, Gerdina H. M. Pijnenborg, Jim van Os, André Aleman, Maaike H. Nauta, Agna A. Bartels-Velthuis

**Affiliations:** 1 Department of Clinical Psychology and Experimental Psychopathology, Faculty of Behavioural and Social Sciences, University of Groningen, Groningen, the Netherlands; 2 Department of Psychotic Disorders, GGZ Drenthe, Assen, the Netherlands; 3 UMC Utrecht Brain Center, Utrecht University, Utrecht, the Netherlands; 4 Department of Neuroscience, University Medical Center Groningen, University of Groningen, Groningen, the Netherlands; 5 Rob Giel Research center, University Center for Psychiatry Groningen, University Medical Center Groningen, University of Groningen, Groningen, the Netherlands; The University of Melbourne, AUSTRALIA

## Abstract

**Aims:**

Theory of Mind (ToM) is often impaired in early and chronic phases of psychosis and it is often suggested that poor ToM is a trait vulnerability for psychosis. The aim of this study was to examine in an adolescent sample whether childhood ToM abilities can predict psychotic experiences over a period of six years and whether this is mediated by social functioning. To examine whether ToM is a specific predictor for psychosis, symptoms of depression and anxiety were also examined.

**Materials and methods:**

A baseline case-control sample (T0: age 7–8 years) with and without auditory vocal hallucinations (AVH) in the general population was assessed after five years (T1: age 12–13 years) on ToM ability (ToM Storybook Frank), and after eleven years (T2: age 18–19 years) on psychotic experiences (Community Assessment of Psychic Experiences; CAPE), depressive and anxiety symptoms (Depression Anxiety and Stress Scale; DASS-21), and social functioning (Groningen Questionnaire on Social Behaviour; GSVG-45). Analyses were conducted on a subsample of 157 adolescents aged 18–19 years (T2) who had data available on ToM ability at T1.

**Results:**

ToM at T1 was not predictive of psychotic experiences after six years (from age 12–13 to age 18–19) and social functioning was also not a mediator. ToM was not associated with psychopathology in general (depressive and anxiety symptoms) over six years (from age 12–13 to age 18–19).

**Conclusions:**

The current study found no evidence for a longitudinal association between ToM ability and psychotic experiences, social functioning, and symptoms of depression and anxiety, in adolescence.

## Introduction

Psychotic disorders often co-occur with deficits in social cognition [1], which are found to contribute considerably to the impairment in social functioning associated with these disorders [2]. Theory of Mind (ToM) is a domain of social cognition [3], and is defined as the ability to represent human mental states or making inferences about someone else’s intentions or emotions [4]. Since Frith (1992) [5] hypothesized that ToM deficits may account for the development of (amongst others) delusions and third-person auditory hallucinations, ToM has been investigated extensively in psychotic disorders [6]. There is evidence that ToM is impaired in multiple phases of psychosis, such as in acute psychosis [7], first episode psychosis [8], individuals at risk for psychosis [9,10] and first-degree relatives of individuals with schizophrenia [7]. In addition, ToM ability often does not necessarily fluctuate with symptoms [10], suggesting that a deficit in ToM may be a trait marker for psychosis rather than a state-related factor [7,11], though not all evidence is consistent with this [12]. It is important to investigate when the ToM vulnerability for psychosis can be ‘detected’ as to examine, as a first step, whether and when early interventions may be possible and effective. Given that psychotic experiences are prevalent in samples of youth [13–15], and may signify a precursor to psychotic disorders [16], it is fruitful to examine whether poorer ToM is associated with psychotic experiences during adolescence.

Currently, there is limited research on ToM ability in association to psychotic experiences in childhood and adolescence. A previous cross-sectional study found that ToM ability at age 12 years was not associated to psychotic experiences [17], whilst another study found that ToM ability at age five years was prospectively associated with definite psychotic symptoms at age 12 years [18]. Whether ToM ability in childhood is associated with psychotic experiences at later ages (18–19 years) in adolescence is a relevant and understudied question. Given that psychotic experiences occurring late in adolescence are more predictive of mental health problems in adulthood [19], answering this question might shed more light on the role of ToM in the development of psychosis.

Impaired social cognition, including ToM ability, has been hypothesized to be the underlying driving source of impaired social functioning in psychotic disorders [4]. So far, the link between ToM and social functioning has been explored mostly in studies with clinical samples. Findings imply that impaired ToM is strongly associated with several domains of social functioning [20]. ToM abilities in patients diagnosed with schizophrenia were positively associated with social functioning [21] and interpersonal skills [22], and negatively associated with socially deviant behaviour [2]. In addition, a positive association between ToM and global functioning in an ultra-high-risk (UHR) sample was found [23], indicating the association may already be present before the first psychotic episode. Despite the emerging evidence that ToM may be a trait marker for psychosis [9], it remains unclear whether a ToM vulnerability for psychosis can be detected during childhood and adolescence. If a ToM deficit represents a vulnerability for psychosis, individuals who later go on to develop schizophrenia may already have poor ToM ability during childhood [24], which could influence their early social interactions with parents and peers. In line with this idea, one might expect children and adolescents with poorer ToM ability to function worse as well, rendering them more liable for psychotic experiences.

Social functioning, which includes the ability to meet societal defined roles such as being a homemaker, worker, student, spouse, family member or friend [25], is commonly impaired in psychotic disorders [4,26]. It has been suggested that a decline in social functioning may be a subclinical marker for psychosis [27,28]. Support for this view comes from studies comparing social functioning of individuals at UHR for psychosis and of first-episode psychosis patients with healthy controls [29,30], demonstrating that social functioning appears to be impaired in early phases of psychotic disorders and even before the first psychotic episode. In fact, a decline in social functioning has been shown to be predictive of a transition to the first psychotic episode in UHR individuals [31]. We hypothesize that an individual with increasing social functioning difficulties may have less positive social experiences and may develop less social skills, potentially rendering an individual more vulnerable to the development of paranoia [32] and delusional ideation [33]. The question remains when this deficit in social functioning first becomes apparent. There is already some evidence that an increase in peer problems and a decline in prosocial behavior precede psychotic experiences during early adolescence [17]. As deficits in ToM may predict impaired social functioning [4] and poor social functioning may predict enhanced psychotic experiences [30], it is warranted to examine whether social functioning potentially (and partially) mediates the relationship between ToM and psychotic experiences.

The current study aims to examine in a general population sample of adolescents whether poorer ToM ability at age 12–13 years is associated with the frequency of psychotic experiences at age 18–19 years. In addition, it will be examined whether social functioning mediates the relationship between ToM ability and the frequency of psychotic experiences. Given that recent studies have noted that risk factors may not be specific for psychosis but predictive of psychopathology in general [34], we will also examine the association between ToM ability and symptoms of depression and anxiety.

## Materials and methods

### Participants

**Fig 1:**
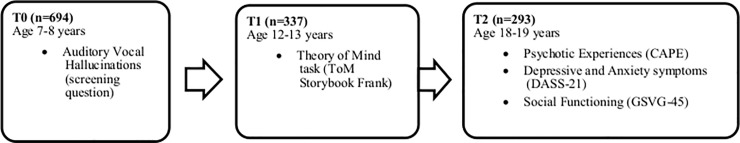
Measurement waves (T0, T1, & T2) of the case-control study. Sample size, age and measures are displayed at each measurement wave. Note that the total sample size studied (n = 157) in the current study differs from the N’s displayed in the table. CAPE, Community Assessment of Psychotic Experiences; ToM, Theory of Mind; DASS-21, Depression Anxiety and Stress Scale; GSVG-45, Groningen Questionnaire on Social Behaviour.

A baseline case-control sample (n = 694) was recruited eleven years earlier from a population-based survey on AVH in almost all 7- and 8-year-old children (n = 3870) in the province of Groningen, the Netherlands [13]. Data from this case-control sample was available at age 7/8 years (T0, [13]), age 12/13 years (T1, [35]) and age 18/19 years (T2, [36]) (Fig 1). For a full description of the samples at baseline and each follow-up study, see previous publications on this sample [13,35,36]. Given that not all participants in the second follow up assessment had completed the ToM task at the first follow up assessment, the current study was conducted on a subsample of 157 adolescents aged 18–19 years (54% of the 293 participants at T2).

### Measures

#### Theory of Mind (ToM)

ToM was assessed at T1 using the ToM Storybook Frank for children aged 10–14 years [37,38]. A validation study in a Danish child and adolescent sample demonstrated that the Storybook Frank task has good psychometric properties [39]. The task consists of multiple domains: first- and second-order false beliefs, deception paradigm, white lie, irony, double bluff and faux-pas. Children are presented with 20 pictures whilst the storybook is read aloud by the interviewer. Children answer 22 ‘test’ and 10 ‘justification questions’. The ‘test’ answers are coded with a 1 (correct understanding of the situation) or 0 (incorrect understanding of the situation), yielding a range of 0 to 22. The ‘justification’ questions (e.g. Why does Frederik think that?) are coded on an ordinal scale ranging from 0 to 1, 2 or 3 (different predefined scale for each tested situation), with a total score ranging from 0 to 25. Scores for each question depend on the level and quality of references made to the thoughts, beliefs, feelings or intentions of the story characters or the child itself. The ‘test’ and ‘justification’ scores are summed together in a total score (maximum score = 47, higher scores indicate better ability). ToM ability at T1 was dichotomized into relatively low (children scoring equal to, or below the mean of 27.19, SD = 4.87) or high ability (children scoring above the mean) in this sample (in line with earlier publications on the T1 sample; 26). The low ToM ability group had a mean ToM ability of 23.57 (SD = 2.89) and the high ToM ability group had a mean ToM ability of 31.54 (SD = 2.74).

#### Psychotic experiences

Psychotic experiences were assessed at T2 with the Community Assessment of Psychic Experiences (CAPE; [40,41]), a 42-item self-report questionnaire to assess the frequency and distress of psychotic experiences in general population samples. The CAPE has previously shown to have good discriminant and convergent validity and good psychometric properties [40,42]. In this study, psychotic experiences were assessed using the frequency of positive psychotic experiences (20 items, rated on a four-point scale: 0 ‘never’, 1 ‘sometimes’, 2 ‘often’ and 3 ‘nearly always’). A total score was created by adding up the 20 frequency items, with a higher score indicating a higher frequency of psychotic experiences.

Given that at least part of the adolescents in this sample were previously selected on auditory vocal hallucinations (AVH) in childhood [13], the analyses in this study will control for this. At baseline (T0), the presence of AVH was assessed with the following question: ‘In the past five years, have you heard one or more voices that only you and no one else could hear?’ (‘yes/no’; [13].

#### Depressive and anxiety symptoms

Symptoms of depression and anxiety were assessed at age 18–19 (T2) with the Depression Anxiety and Stress Scale (DASS-21; [43]; Dutch translation; [44]). The DASS is a 21-item self-report measure of symptoms of anxiety, depression and stress, which has shown to have good reliability and validity in a sample of non-clinical adolescents [45]. Each of the three subscales of the DASS-21 contains seven items. A 4-point severity scale measures the extent to which each state has been experienced over the past week. For comparability with the 42-item DASS, scores were doubled in accordance with the makers of the scale [43]. A sum score was created for the depression subscale and for the anxiety subscale, by adding the scores of all seven items of each subscale, with a higher score indicating a higher severity of symptoms of depression and anxiety.

#### Social functioning

Social functioning was assessed at T2 with the 45-item self-report questionnaire Groningen Questionnaire on Social Behaviour (in Dutch: de Groningse Vragenlijst over Sociaal Gedrag (GSVG-45; [46]). Ten items enquiring about the participants’ functioning as a parent were omitted as our sample consisted of adolescents. The remaining 35 items assess functioning with parents (“Lately I have avoided (one of my) parents”), a significant other (“I discussed my personal issues with my boyfriend/girlfriend”), friends/acquaintances (“I enjoyed spending time with my friends/acquaintances”), education (“I was late at school (in the classroom/lecture hall)”), vocation (“I was able to finish my work on time”), household chores (“I found it difficult to adhere to the daily rules of the household”), and hobbies (“I was able to relax in my free time”), each consisting of five items with a 4-point scale (‘never’, ‘sometimes’, ‘often’, ‘always’). A mean score of positive responses (scores 1–4) was created for the overall social functioning scale (max 35 items), with a higher mean score indicating better social functioning. The social functioning items showed good internal consistency in this study (Cronbach’s alpha: 0.83). Due to a technical error, three questions pertaining to the ‘hobby’ domain were not administered, resulting in a total of 32 questions for the overall social functioning scale.

#### Education

Education was assessed at T2 in a separate sociodemographic questionnaire (see previous publication, [36]) assessing the level of current education. Answer options included (1) primary education, (2) lower vocational education, (3) higher general secondary education (4) pre-university secondary education, (5) intermediate vocational education, (6) higher vocational education and (7) university education. In line with a previous study using this sample [36], education level was dichotomized into low levels of education (options 1, 2, 3, and 5) and high levels of education (options 4, 6 and 7).

### Procedures

This study was approved by the Medical Ethical Committee of the University Medical Center Groningen, the Netherlands (ABR number NL42619.042.12). For the first follow-up, written informed consent was obtained from both parents and children. For the child, the explanation of the study was adapted to their developmental level. The ToM task was administered by trained interviewers at the child’s home in absence of the parent(s) [35]. For the second follow-up, participating adolescents sent a written informed consent form by post. After receipt of the consent, an email was sent with a link to online questionnaires, assessing demographic information, psychotic experiences, psychopathology and social functioning [36]. For all assessment points, participants had (and were informed of) the right to withdraw from the study at any point.

### Statistical analyses

Statistical analyses were conducted using SPSS 23 [47]. Significance tests were two-tailed with alpha set at 0.05. In order to explore whether social functioning at T2 mediates the possible association between ToM at T1 and the frequency of psychotic experiences at T2, the computational procedure PROCESS [48] was utilized. A ‘simple mediation model’ was computed, which includes the direct effect of ToM ability (T1) on the frequency of psychotic experiences (T2), with the indirect effect operating through social functioning (T2). The direct and indirect effects of ToM (T1) on the frequency of psychotic experiences (T2) were obtained from three linear regression models [49,50]. The Sobel test was used as an inferential method to test hypotheses about the indirect effect [51]. In addition, bias-corrected bootstrap confidence intervals using 10,000 bootstrap samples are computed. Mediation is established if the confidence interval for an indirect effect does not include zero.

To examine specificity of ToM ability for psychotic experiences, two longitudinal linear regression models were computed to examine the predictive ability of ToM at T1 on symptoms of depression and anxiety at T2. All analyses in this study were adjusted for AVH at baseline (T0), and educational level at T2 (dichotomized into low and high).

## Results

The 157 participants at T2 had a mean age of 18.9 years (SD = 0.35, range 18.2–19.9). At baseline, 76 reported AVH and 81 did not (*X*^2^ = 0.05, p = 0.83). Significantly more females participated in this study (57.3%) compared to males (*X*^2^ = 12.2, p = 0.001), but there were no significant differences between males and females in ToM ability at T1 (males: M = 26.60, SD = 5.01, females: M = 27.18, SD = 5.01, t(155) = .519, p = .605) and in the frequency of psychotic experiences at T2 (males: M = 4.12, SD = 4.42, females: M = 4.44, SD = 4.36, t(149) = .630, p = .530).

Data on ToM ability at T1 in addition to CAPE scores at T2 was available for 52% of the 293 T2 participants (n = 151; 6 individuals did not complete the CAPE). Missing values on T1 ToM ability were a result of: (i) the ToM task at T1 being optional and some participants choose not to partake in the assessment (N = 78 of T1 adolescents (23%), resulting in n = 54 of T2 adolescents (18%)), and (ii) 88 participants (30%) in the current follow-up not partaking at T1 but having the option to re-enter the study at T2. Participants at T2 who had data of the ToM task at T1 did not significantly differ in the frequency of psychotic experiences or average social functioning, from participants at T2 who did not take part at the ToM task at T1.

Data on baseline AVH, ToM ability at T1, and depressive and anxiety symptoms, the frequency of psychotic experiences and average social functioning at T2, can be found in Table 1.

**Table 1:** Data on baseline AVH, ToM ability at T1, and depressive and anxiety symptoms, frequency of psychotic experiences and social functioning at T2 (n = 157).

		Frequency (%) or Mean (S.D.)	Range
(T0)			
AVH	No	81 (51.6)	
	Yes	76 (48.4)	
(T1)			
ToM ability	Low	82 (52.2)	
	High	75 (47.8)	
(T2)			
Educational level	Low	66 (49.6)	
	High	67 (50.4)	
Frequency of Psychotic Experiences		4.09 (4.59)	0–60
Depressive symptoms		6.11 (6.32)	0–42
Anxiety symptoms		4.75 (4.59)	0–42
Social Functioning		3.31 (.33)	1–4

Note

AVH, Auditory Vocal Hallucinations; ToM, Theory of Mind.

### Mediation analysis: ToM ability at T1 predicting the frequency of psychotic experiences at T2, as mediated by social functioning at T2

See Table 2 for a summary of the mediation analysis computed with PROCESS. It can be seen that the direct effect of ToM ability at T1 on psychotic experiences at T2 was not significant. The 95% confidence interval of the bootstrap results revealed that the indirect effect of ToM ability at T1 on psychotic experiences at T2 through social functioning at T2 included zero, indicating that social functioning was not a mediator of the association between ToM ability and psychotic experiences. This was supported by the Sobel test, demonstrating no mediation in the model (z = 1.00, p = 0.32). Notably, the combination of all variables in the model (ToM ability, social functioning and control variables) explained a significant proportion of variance in the frequency of psychotic symptoms (R^2^ = 16%, F(3, 147) = 5.99, p = 0.001).

**Table 2:** Mediation analysis: ToM ability at T1 predicting the frequency of psychotic experiences at T2, as mediated by social functioning at T2 (n = 151).

	B	B S.E.	t	p-value	95% Lower	C.I. Upper
Effect of ToM T1 on social functioning T2	-.07	.06	-1.06	.29	-.19	.06
Effect of social functioning T2 on psychotic experiences T2	-3.91	.96	-4.08	.00	-5.81	-2.01
Effect of ToM T1 on psychotic experiences T2	1.14	.66	1.72	.09	-.17	2.45
Bootstrap result for indirect effect of ToM T1 on psychotic experiences T2 through social functioning T2	0.26	0.30			-.14	1.09
R^2^ = 16% Sobel test: z = 1.00, p = 0.32						

Note.

All analyses are corrected for voice hearing at baseline and educational level at T2.

ToM, Theory of Mind; T1, first follow-up at age 12–13 years; T2, second follow-up at age 18–19 years.

### Specificity of ToM for psychosis: the longitudinal relationship between ToM ability at T1 and symptoms of depression and anxiety at T2

See Table 3 for a summary of the two regression models. ToM ability at T1 was not significantly associated with symptoms of depression or anxiety at T2. The variance of symptoms of depression and anxiety that was explained by the model, was small and insignificant (depression: R^2^ = 3%, F(3, 147) = 1.41, p = 0.24, anxiety: R^2^ = 3%, F(3, 147) = 1.46, p = 0.23).

**Table 3:** Longitudinal linear multiple regression models to predict depressive and anxiety symptoms at T2, with ToM ability at T1 (N = 151).

	Depression							Anxiety						
						95% C.I.							95% C.I.	
	B	SE B	β	t	p	Lower	Upper	B	SE B	β	t	p	Lower	Upper
Constant	3.63	2.32		1.57	.12	-.95	8.22	5.23	1.70		3.08	.00	1.86	8.60
Education	-.64	1.16	-.05	-.56	.58	-2.94	1.65	-1.26	.85	-.13	-1.47	.14	-2.94	.44
AVH (T0)	1.17	1.14	.09	1.03	.31	-1.08	3.42	1.08	.84	.11	1.30	.20	-.57	2.74
ToM ability T1	2.04	1.16	.16	1.75	.08	-.27	4.34	.78	.86	.08	.91	.37	-.92	2.47
R^2^ of model	3%							3%						

Note.

ToM, Theory of Mind; T2, second follow-up at age 18–19 years; T1, first follow-up at age 12–13 years; AVH, Auditory Vocal Hallucinations; T0, baseline at age 7–8 years.

### Post-hoc exploration: 10% of individuals with lowest scores on ToM ability at T1

To investigate whether a specific subsample with the lowest ToM abilities are at risk for psychotic experiences, we conducted a post-hoc exploration. The group with the 10% lowest scores (mean ToM T1 ability: 20 or lower) were compared with the rest of the group (top 90% of ToM T1 scores) on psychotic experiences at T2. A Mann Whitney *U* test showed no significant difference between the group of 10% lowest scores (Mdn = 2) and the remaining scorers on the ToM task at T1 (Mdn = 3), on the frequency of psychotic experiences at T2 (*U* = 1346, p = .11). It was also examined whether there were differences between the group with the lowest 10% of scores (Mdn = 3.47) and the group of remaining scorers on the ToM task at T1 (Mdn = 3.31), on social functioning at T2. No significant differences were found (*U* = 800, p = .10).

### Post-hoc exploration: Continuous measure of ToM ability at T1

To investigate whether the dichotomization of ToM ability at T1 was responsible for the lack of an association between ToM ability at T1 and the frequency of psychotic experiences at T2, a post-hoc exploration was conducted using the sum score of ToM ability at T1. See Table 4 for a summary of the regression model. The continuous measure of ToM ability at T1 was also not significantly associated with the frequency of psychotic experiences at T2.

**Table 4:** Longitudinal linear multiple regression model to predict the frequency of psychotic experiences at T2, with a continuous measure of ToM ability at T1 (N = 151).

	Frequency of psychotic experiences						
						95% C.I.	
	B	SE B	β	t	p	Lower	Upper
Constant	2.81	1.96		1.44	.15	-1.07	6.69
Education	-1.37	.70	-.18	-1.96	.05	-2.75	.02
AVH (T0)	-.07	.68	-.01	-.10	.92	-1.41	1.28
Sum score of ToM ability T1	.11	.07	.15	1.69	.10	-.02	.25
R^2^ of model	4%						

Note.

ToM, Theory of Mind; T2, second follow-up at age 18–19 years; T1, first follow-up at age 12–13 years; AVH, Auditory Vocal Hallucinations; T0, baseline at age 7–8 years.

## Discussion

In line with the idea that poor ToM ability may represent a vulnerability for developing psychosis [7,11], the current study examined in a general population sample of adolescents whether poorer ToM ability at age 12–13 years was associated with the frequency of psychotic experiences at age 18–19 years. In addition, it was examined whether social functioning at age 18–19 years mediates this relationship. Contrary to our expectations, we did not find evidence that poorer ToM ability in childhood was longitudinally associated to increased psychotic experiences in adolescence over a period of six years. This was confirmed in a post-hoc exploration when examining the bottom 10% of scorers on the ToM task, again establishing no evidence for increased psychotic experiences. Social functioning was therefore not identified as a mediator between ToM ability and psychotic experiences. Similarly, ToM ability did not predict symptoms of anxiety or depression over six years’ time. The findings imply that in the current adolescent general population sample, ToM ability was not a vulnerability factor for psychotic experiences, social functioning, or for depression and anxiety.

Given that the literature is quite consistent in the hypothesis that ToM ability might represent a vulnerability factor for the development of psychosis [7,9,11], we were surprised not to find an association between early ToM ability at age 12–13 years and the frequency of psychotic experiences at age 18–19 years. On the basis of our findings, we speculate that the group of poorer scorers on the ToM task (those scoring equal to, or below the mean; roughly half of the sample) may not be a proxy of an at ‘risk’ or ‘vulnerability’ group, and therefore is not impaired enough to detect differences in psychotic experiences. In our post-hoc exploration we selected 10% of the lowest scorers on the ToM task, which we considered to have impaired levels of ToM ability (similar to ToM scores in a high functioning autism spectrum disorder group; [39]). However, this impaired sub-group did not report a higher frequency of psychotic experiences in comparison to adolescents who performed higher on the ToM task. Our findings are supported by a recent study [52] indicating that an increasing developmental (verbal and non-verbal) cognitive deficit between infancy and adulthood is only present for individuals who develop a psychotic disorder, with only weak evidence for individuals with psychotic experiences. As most adolescents in this sample will not develop a psychotic disorder (prevalence in general population samples 1%, [53]; prevalence for adolescents with psychotic experiences 7.5%, [54]), it may be difficult to detect a ToM vulnerability for psychosis in this adolescent sample. Larger samples with a longitudinal design may yield more power to examine the development of the ToM vulnerability for psychosis during childhood and adolescence. In addition, including a later follow-up assessment including clinical diagnostic information could verify our speculations that perhaps only those adolescents who go on to develop a psychotic disorder will have demonstrated early signs of a ToM vulnerability in adolescence.

It is important to emphasize that that the current findings are based on a general population sample of adolescents with mild psychotic experiences. Although the current sample was recruited as a case-control cohort from the general population on auditory vocal hallucinations 11 years earlier, most adolescents no longer heard voices at age 18–19 years (only 15; [36] and were therefore considered to meet similar levels of psychotic experiences as in a random sample from the general population. Indeed, the average number of psychotic experiences in the current sample was lower than an UHR of psychosis sample ([55]; our sample: 1.2, vs. UHR sample: 1.6–1.9) and therefore cannot be viewed as meeting clinical levels. Moreover, although psychotic experiences have the ability to predict psychotic disorders [54], the majority of psychotic experiences disappear over time [14,36]. Therefore, perhaps this sample was not suitable to detect a ToM vulnerability for psychotic experiences. When comparing our findings to earlier studies, it has previously been found that ToM ability of children at age 12 was not associated with psychotic experiences cross-sectionally [17]. On the other hand, ToM ability at age five years has been found to be predictive of definite (more clinical) psychotic symptoms at age 12 years [18]. This is supportive of the idea that ToM ability may not be related to psychotic experiences in adolescence more broadly, but only to clinical psychotic symptoms or disorders.

In our study, ToM ability at age 12–13 years was not associated to social functioning at age 18–19 years, and social functioning was not a mediator between ToM ability and the frequency of psychotic experiences. When examining adolescents who scored in the bottom 10% of the sample on ToM ability at T1, there was no evidence for poorer social functioning in comparison to adolescents who scored higher on the ToM task. We therefore found no evidence that ToM ability was longitudinally associated with social functioning in this general population sample of adolescents. In clinical samples it is often found that ToM is an important predictor for several domains of social functioning (e.g. [4]. However, a study on a young UHR sample found that although ToM deficits were present, they were not associated with social functioning with parents (specifically experiences of the caregiving relationship; [56]. Similarly, a recent study found that ToM was not predictive of social functioning in individuals with recent-onset psychosis [57]. We speculate that ToM ability only affects social functioning when ToM is below a certain threshold, one that is only reached during more acute phases of psychosis [21]. Above this threshold, an individual may be able to compensate poorer ToM ability with other factors (e.g. cognitive abilities, motivational strategies or personality) in order to retain effective social functioning.

The current study has some limitations. First, the current sample was a sub-sample of a larger sample, of whom not all participants took part in the previous ToM task. We therefore have an opportunistic sample that may be biased as we only studied the adolescents who were willing and able to participate in a ToM task. However, when comparing adolescents who did and did not participate in the ToM task on psychotic experiences and social functioning, we did not find any significant differences. Second, it is possible that some participants were or had been in care for mental health problems, as their health care consumption was not assessed at the time. However, this was purposely done in order to avoid pathologizing auditory hallucinations. Third, perhaps an association between ToM ability and the frequency of psychotic experiences would become evident when changing the threshold of 10% to an even lower level (e.g. 5% lowest scorers). However, larger sample sizes are required to have enough power to detect such differences. In addition, one could speculate that the dichotomization of ToM ability was responsible for a loss in data and therefore the null-findings in this study. However, when checking our analysis using continuous measures in the post-hoc exploration, the same (null-) result was found. Fourth, the ToM task has been validated in a Danish sample and in a shorter form [39], thus we cannot confirm that the psychometric properties will be identical in the current sample. Fifth, the utilized sub-sample consisted of significantly more females than males. Given that gender differences exist in how psychosis develops [58], future research should aim to include equal numbers of males and females in their study. Last, in contrast to the first assessments (at T0 and T1) in this sample, assessments at T2 took place in a self-report form in an online format. This may limit comparability of findings to previous assessment points in the current longitudinal sample and other studies examining similar questions. Nevertheless, we also believe there is a benefit in online and self-report assessments, as there may be a lower chance of social desirability bias and perceived stigma in the reporting of psychotic experiences.

Future research should examine the same associations using different domains of social cognition, such as emotion recognition, given that other social cognitive domains are also impaired in early phases of psychosis [59] and are consistently associated with social functioning [4]. This might be especially relevant as a review [59] demonstrated that deficits in emotion recognition were found to be consistently impaired in first episode psychosis, especially in lower level abilities (e.g. face and voice emotion recognition), whereas some domains of ToM remained intact (first-order ToM abilities). Therefore, it is possible that deficits in social cognitive domains other than ToM, such as emotion recognition, are more sensitive to be detected before the first psychotic episode. So far there are only few studies which have examined whether emotion recognition is prospectively associated with psychotic experiences in childhood and adolescence. One of these studies [60] did not find an association between facial emotion identification at age 8 years and psychotic experiences at age 11 years. Given that emotion recognition, a lower-level social cognitive ability, may not be associated with psychotic experiences in childhood, it may also be unlikely that ToM ability (a higher-level social cognitive ability) is associated with psychotic experiences. To explore these speculations further, research is required to prospectively examine multiple domains of social cognition, and their inter-relations, in association with psychotic experiences in childhood and adolescence.

To conclude, although it has been suggested that a deficit in ToM may be a vulnerability marker for psychosis, the current study found no longitudinal evidence for an association between ToM ability and the reporting of psychotic experiences in adolescence. It was also demonstrated that ToM ability was unrelated to social functioning, and to symptoms of depression and anxiety in adolescence. Based on our findings and in the context of the presented literature [17,18,52], we speculate that ToM deficits may only be associated with clinical psychotic symptoms or psychotic disorders, and not psychotic experiences more broadly.
